# The influence of radiotherapy techniques on the plan quality and on the risk of secondary tumors in patients with pituitary adenoma

**DOI:** 10.1186/s12885-020-6535-y

**Published:** 2020-02-03

**Authors:** Marius Treutwein, Felix Steger, Rainer Loeschel, Oliver Koelbl, Barbara Dobler

**Affiliations:** 1 Department for radiotherapy, Regensburg University Medical Center, Regensburg, Germany; 2 Faculty of computer science and mathematics, Ostbayerische Technische Hochschule, Regensburg, Germany

**Keywords:** Pituitary adenoma, Treatment planning, Secondary malignoma risk, Flattening filter free

## Abstract

**Background:**

This planning study compares different radiotherapy techniques for patients with pituitary adenoma, including flatness filter free mode (FFF), concerning plan quality and secondary malignancies for potentially young patients. The flatness filter has been described as main source of photon scatter.

**Material and methods:**

Eleven patients with pituitary adenoma were included. An Elekta Synergy™ linac was used in the treatment planning system Oncentra® and for the measurements. 3D plans, IMRT, and VMAT plans and non-coplanar varieties were considered. The plan quality was evaluated regarding homogeneity, conformity, delivery time and dose to the organs at risk. The secondary malignancy risk was calculated from dose volume data and from measured dose to the periphery using different models for carcinoma and sarcoma risk.

**Results:**

The homogeneity and conformity were nearly unchanged with and without flattening filter, neither was the delivery time found substantively different. VMAT plans were more homogenous, conformal and faster in delivery than IMRT plans. The secondary cancer risk was reduced with FFF both in the treated region and in the periphery. VMAT plans resulted in a higher secondary brain cancer risk than IMRT plans, but the risk for secondary peripheral cancer was reduced. Secondary sarcoma risk plays a minor role. No advantage was found for non-coplanar techniques. The FFF delivery times were not shortened due to additional monitor units needed and technical limitations. The risk for secondary brain cancer seems to depend on the irradiated volume. Secondary sarcoma risk is much smaller than carcinoma risk in accordance to the results of the atomic bomb survivors. The reduction of the peripheral dose and resulting secondary malignancy risk for FFF is statistically significant. However, it is negligible in comparison to the risk in the treated region.

**Conclusion:**

Treatments with FFF can reduce secondary malignancy risk while retaining similar quality as with flattening filter and should be preferred. VMAT plans show the best plan quality combined with lowest peripheral secondary malignancy risk, but highest level of second brain cancer risk. Taking this into account VMAT FFF seems the most advantageous technique for the treatment of pituitary adenomas with the given equipment.

## Background

Radiotherapy of pituitary adenomas is often applied as postoperative therapy of tumors that cannot be removed completely. For over one decade patients with pituitary adenoma have been treated primarily with two parallel opposed fields or a three field technique [1–7]. Only a few recent publications describe the application of intensity modulated radiotherapy (IMRT) or volumetric modulated arc (VMAT) technique in this region [8–10], none of them mentioning the flatness filter free (FFF) mode. FFF is applicable in combination with fluence modulating techniques like IMRT or VMAT. In this mode a considerably higher dose rate is achieved by omitting the flatness filter. This planning study compares different plans for patients with pituitary adenoma. The plans were optimized using both modes: flattened beam (FB) and FFF. The flatness filter has been described as the main source of photon scatter in the treatment head [11]. Additional peripheral dose resulting from this contribution has been confirmed in some publications referring to other entities [12–18] and increases the risk for secondary malignancies. Pituitary adenomas represent about 10% of all intracranial tumors [19]. The incidence increases over the years, starting at about an age of 10 years and has a possible decline in high age [20]. Therefore, the risk for secondary malignancies should be considered. The aim of this study is to evaluate statistically significant differences for FB and FFF plans regarding the plan quality and the risk for secondary malignancies in the treated region and the periphery. Plans with two and three fixed beams were taken as reference.

## Material and methods

### Patients

Data sets of 11 patients (five female, six male) with pituitary adenoma were used for this retrospective planning study. All patients have got a cranial X-ray CT scan in supine positioning with the head in neutral position. The head was immobilized using thermoplastic mask systems. The CT scans were fused with the pretherapeutic cranial MRI (contrast enhanced T1-weighted sequence). GTV, CTV and PTV as well as organs at risk were delineated using the treatment planning system (TPS) Oncentra® External Beam v4.5 (Nucletron®, an Elekta company) on all axial slices. The GTV included the macroscopic (residual) tumor volume. The CTV was based on GTV extended for the resection cavity in postoperative cases. The PTV was defined by CTV plus an isotropic margin of 3–5 mm, depending on setup error and reproducibility of positioning.

### Linear accelerator

We used a linear accelerator (linac) of type Elekta Synergy™ with Agility™ head (Elekta AB, Stockholm, Sweden) operated by the desktop software Integrity 3.1 and record and verify system Mosaiq 2.50. The head is equipped with 80 interdigitating leaf pairs, projecting a leaf width of 5 mm to the isocenter. For all plans 6 MV photons were used. The beam quality of both modes FB and FFF has been shown to be equivalent for this machine type [21, 22]. The linac offers a maximum dose rate of 550 MU per minute in FB mode and 1700 MU per minute in FFF.

### Treatment planning

The planning was performed with TPS Oncentra® External Beam using the collapsed cone algorithm. A grid size of 2 mm has been chosen. Variable gantry speed with a set maximum value of 6.0 degree per second is supported by the software. The variable dose rate was set to a minimum value of 20 MU per minute. A dynamic and static minimum leaf gap of 1.0 cm had to be observed. The optimizer module in Oncentra® used the step-and-shoot algorithm for IMRT optimizations [23]. This module has been developed by RaySearch Laboratories (Stockholm, Sweden) and therefore has the same roots as the SmartArc module in Pinnacle^3^ TPS (Philips, Amsterdam, Netherlands) and the proprietary development RayArc module in RayStation TPS.

The objectives for the PTV were set to a minimum dose of 49.4 Gy and a maximum dose of 51.4 Gy in 28 fractions, aiming for a fraction dose of 1.8 Gy. A uniform dose objective to 50.4 Gy was added to improve the homogeneity. Further objectives were set for the following organs at risk (OAR) (Table 1) according to [24–26]: brain, brainstem, chiasm, both lenses, bulbs, lacrimal glands, and parotids. Additionally, the surrounding dose fall-off objective has been applied to shape the dose gradient from the PTV into the normal tissue [27]. It supports an improvement of the conformity. The same set of dose volume objectives has been used for all plans to get comparable results [12, 18, 28, 29]. The aim of this set was to keep the risk for the OARs on an acceptable level, but to leave freedom for the optimizer to achieve good conformity and homogeneity. In both modes (FB and FFF) IMRT plans with nine equispaced coplanar fields were generated; in a second variant a tenth non-coplanar field was added. Similarly two different VMAT plans were optimized: one single arc rotation (182°-178°), and the second with an added half rotation in the sagittal patient plane (0°-180°).

**Table 1:** Treatment planning objectives

Regions of interest	Dose level in Gy	Volume in %	Dose volume objective type	Weight
Brain	20	20	Maximum dose volume	300
	30	10	Maximum dose volume	300
	40	5	Maximum dose volume	300
Brainstem	51.4	0	Maximum dose	500
Chiasm	50	0	Maximum dose	300
Lens	15	0	Maximum dose	30
Bulb	35	50	Maximum dose volume	100
Lacrimal gland	20	50	Maximum dose volume	30
Parotid	30	50	Maximum dose volume	100
Outline	51.4	0	Maximum dose	5000
	49.4–25.0		Surrounding dose falloff, distance 1 cm	3000
PTV	49.9	100	Minimum dose	3000
	51.4	0	Maximum dose	3000
	50.4		Uniform dose	1000

The average dose to the PTV was set to 100% after the optimization process. An average dose value in a range of 50.4 Gy ± 0.8 Gy - which represents an interval of about 1.5% around the target value – was accepted in the sense of a dose prescription according to ICRU 83 [30]. No rescaling has been performed as this would have affected the dose to the normal tissue and organs at risk which are also part of the optimization process [31].

### Plan evaluation

The following parameters were evaluated: the average dose to the PTV D_av_^PTV^, homogeneity index HI, and the conformity index CI. For HI the definition of ICRU report 83 [30] was used: HI = (D_2%_^PTV^ – D_98%_^PTV^)/D_av_^PTV^ with D_2%_^PTV^ and D_98%_^PTV^ as dose to 2 and 98% of the PTV. CI was defined according to Paddick [32]: CI = TV_49.4 Gy_^2^/(V_49.4 Gy_ x V^PTV^). TV_49.4 Gy_ is the volume within the PTV which receives at least 49.4 Gy, V_49.4 Gy_ is the volume enclosed by the corresponding isodose within the complete patient contour, and V^PTV^ is the volume of the PTV. For all plans the observance of the objectives for the OAR was investigated and regarded as criterion of acceptability.

### Measurements

**Fig. 1:**
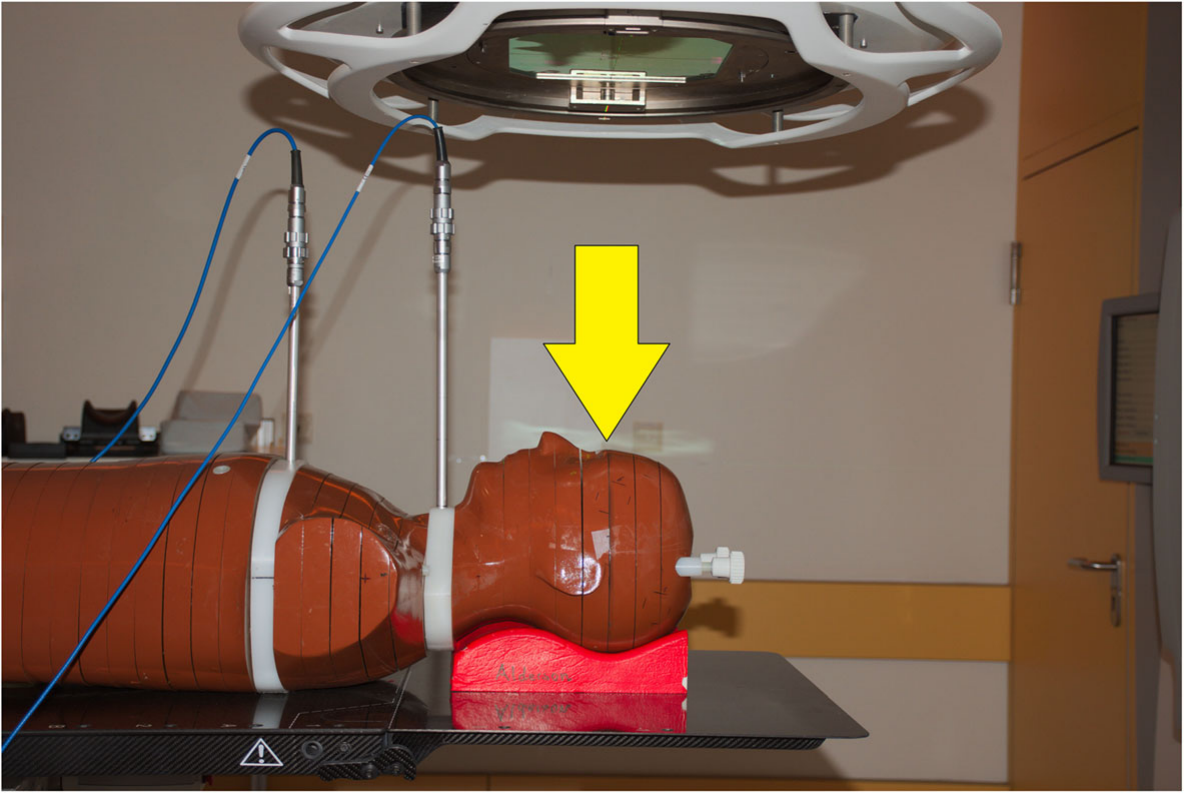
Setup of the Alderson phantom for the peripheral dose measurements. The ionization chambers are inserted in the white slabs. The arrow indicates the isocenter plane

The evaluation of the peripheral dose (PD) was performed using the upper part of a male Alderson phantom (RSD Inc., Long Beach, CA, USA) (Fig. 1). Two slabs of the phantom were replaced by copies of PA material with bores for ionization chambers. The first chamber in a distance of 16.3 cm from isocenter corresponded to the position of the thyroid gland, the second in a distance of 30.3 cm in the upper thoracic region corresponded to an esophageal position. The dose to these points, PD^thyr^ and PD^esoph^ has been measured with chambers type M30016 (0.3 cm^3^) and M23331 (1.0 cm^3^), respectively, both connected to Unidos dosimeters (PTW, Freiburg, Germany). It is reasonable to assume an uncertainty of 5% for these measurements considering the statistical uncertainty, the positioning inaccuracy and the calibration of the detector for megavoltage beam quality [33].

**Fig. 2:**
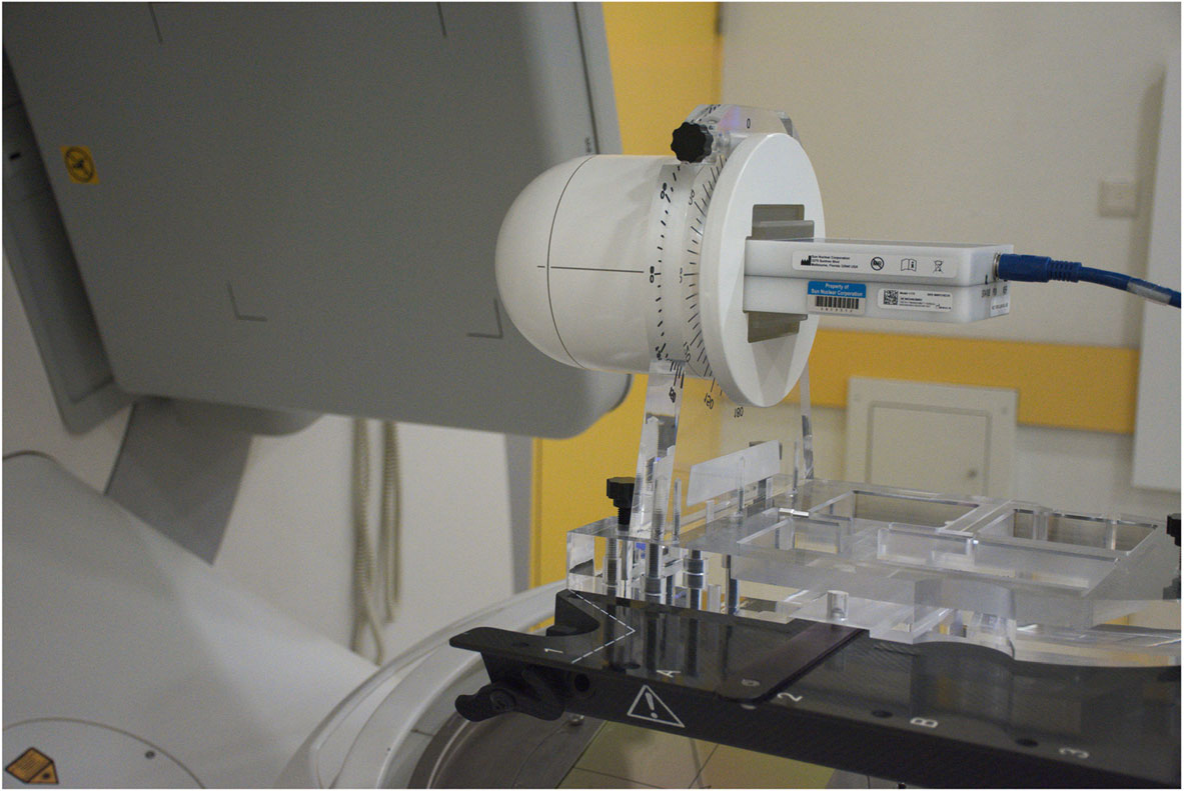
Setup for the plan verification with SRS MapCHECK™ array inserted in the StereoPHAN™ phantom

The plan verifications were accomplished with the SRS MapCHECK™ array and StereoPHAN™ phantom equipment (Fig. 2) in combination with the SNS Patient™ software version 8.1 (Sun Nuclear Corporation, Melbourne, FL, USA). The array is a rather new developed device. Its size is 77 × 77 mm^2^, the diodes have a spacing of 2.47 mm. The active detector area is 0.48 × 0.48 mm^2^. The signals are sampled with a frequency of 20 Hz. Gantry angles are analyzed by the angular dependency of two opposing detector planes. Therefore, no gantry angle sensor is necessary. The phantom has already been used with other modular inserts for films [34] and ionization chambers. The system is especially designed for small volumes.

We applied a hybrid plan verification as described for other systems [35–37] transferring the patient plan unchanged to the phantom and performing a dose calculation on the phantom with a dose grid of 1 mm. A CT scan of the phantom has been offered by the manufacturer, slices in 1 mm distance; a uniform relative mass density of 1.2 has been assigned to the external structure as recommended. The array was positioned horizontally in the isocenter plane.

The software assists a cross-calibration procedure. Offered shifts of the measured profiles relative to the calculated ones for best coincidence were accepted, but were clearly smaller than 1.0 mm. The measured and calculated dose distributions were evaluated by the gamma index [38] with a dose tolerance of 3% referring to the maximum dose and a distance to agreement of 3 mm. The area of the evaluation was confined to dose values above 10% of the dose maximum [39].

As an additional plan quality parameter the delivery time (DT) was recorded from pressing the beam on button until the last beam off.

### Secondary malignancy risk

The calculations for the risk of secondary malignancies use the models presented by Schneider et al. [40]. Their work combines the data of the Japanese A-bomb survivors [41] and secondary cancer data of Hodgkin’s patients from a Western population [42]. The excess absolute risk (EAR) describes the risk of malignancy incidence after irradiation. It is expressed as absolute difference of the number of malignancies in comparison to an untreated control group. Commonly it is given per 10.000 persons per year. It is a function of the dose *d*, the sex *s*, the age at exposure *e* and the attained age *a*.

$$ EAR\left(d,s,e,a\right)=\mu \left(s,e,a\right)\times f(d) $$

In the present work gender averaged values are used. Therefore, the parameter *s* can be neglected. Using the tables given in [40], the EAR can be calculated from the dose volume data from the TPS for different organs of volume V_T_:

$$ {EAR}^{org}=\frac{1}{V_T}\sum \limits_iV\left({D}_i\right)\times {\beta}_{EAR}\times RED\left({D}_i\right)\times \mu \left(e,a\right) $$

The summation is performed over all voxels of the organ with dose entry *D*_*i*_*. μ* can be used to calculate the risk for different ages (*e*: age at exposition, *a*: attained age):

$$ \mu\ \left(e,a\right)=\mathit{\exp}\left(\ {\gamma}_e\left(e-30\right)+{\gamma}_a\ \ln \left(\frac{a}{70}\right)\right) $$

The parameters *γ*_*e*_ and *γ*_*a*_ were derived by Preston et al. [41]. For our calculations we have chosen *e = 35* years, which is close to the mean age given by Yamanaka et al. [43] (37 years) and corresponds to the mean age minus one standard deviation by Jiang et al. [44]. The attained age was set to *a = 70* years.

*β*_*EAR*_ is the initial slope, the risk equivalent dose *RED* the dose dependent part, for which Schneider et al. present different models for carcinoma induction:

- The mechanistic model which considers cell killing and fractionation effects
- The bell-shaped dose response model which neglects any repopulation or repair effect
- The plateau model with full repopulation or repair

The mechanistic model is given by the form

$$ RED(D)=\frac{e^{-\alpha \hbox{'}D}}{\alpha \hbox{'}R}\ \left(1-2R+{R}^2{e}^{\alpha \hbox{'}D}-{\left(1-R\right)}^2{e}^{\frac{\alpha \hbox{'}R}{1-R}D}\right) $$

assuming a fractionated treatment schedule of single fractions with dose *d* up to a total dose *D*. *α’* has been derived from the linear quadratic model, assuming *α/β* = 3 Gy for all tissues.

$$ \alpha \hbox{'}=\alpha +\beta d $$

*R* is the repopulation and repair parameter and equals 0 for no and 1 for full repair or repopulation. The bell-shaped model is got in the limit of *R* to 0:

$$ RED(D)=D\ {e}^{-\alpha \hbox{'}D} $$

In the limit of R to 1 the plateau model is described:

$$ RED(D)=\left(1-{e}^{-\alpha \hbox{'}D}\right)/\alpha \hbox{'} $$

The authors emphasize that there is only little knowledge yet about the dose-response relationships in the investigated dose range [40]. The data could not be fitted by all models for all organs, not even the most complex mechanistic model. Therefore, we performed our calculations for all three models.

At last we also applied the model for sarcoma induction of bone and soft tissue. The formula is quite similar to the mechanistic model for carcinoma induction with one additional term:

$$ RED(D)=\frac{e^{-\alpha \hbox{'}D}}{\alpha \hbox{'}R}\ \left(1-2R+{R}^2{e}^{\alpha \hbox{'}D}-{\left(1-R\right)}^2{e}^{\frac{\alpha \hbox{'}R}{1-R}D}-\alpha \hbox{'} RD\right) $$

We confined our calculations to an intermediate repopulation and repair effect with *R = 0.5*. *β*_*EAR*_, *γ*_*e*_, and *γ*_*a*_ are given in Table 2 for the investigated organs.

**Table 2:** Initial slope and age modifying parameters for EAR calculation [40]

Organ at risk	*β*_*EAR*_	*γ*_*e*_	*γ*_*a*_
Brain	0.51	−0.024	2.38
Soft Tissue	0.60	−0.013	−0.56
Bone	0.20	−0.013	−0.56
Thyroid	–	−0.046	0.6

The two peripheral dose points were situated in the low dose region. It has been shown by Preston et al. [41] that for these points up to a total dose of 2 Gy the simple linear model is applicable.

$$ {EAR}^{org}={\beta}_{EAR}\times D\times \mu \left(e,a\right) $$

The factors *β*_*EAR*_ for the selected OAR were taken from this publication and applied according [40]. They are given per 10.000 persons per year and Gy as 1.2 for the thyroid gland and 0.58 for the esophagus. For the esophagus no age dependency has been found. The age correction factor *μ(e,a)* for secondary cancer risk in the thyroid gland was calculated with γ_e_ and γ_a_ from Table 2. For this calculation the measured point dose has been taken representative for the whole organ.

### Statistics

An a priori power analysis has been performed to determine the sample size using the software G*Power version 3.1.9.2 [45, 46]. We set α = 0.05, power (1-β) = 0.8, and the effect size to 1.0. The Wilcoxon signed-rank test for paired samples was chosen as statistical test, as it does not require a normal distribution of the variables. Taking various parent distributions into account (normal, Laplace, logistic) the maximum sample size of *N* = 11 for a normal distribution was selected.

IBM® SPSS® Statistics v23 (IBM Corporation, Armonk, NY, USA) was applied to perform the Wilcoxon test with a significance level of 0.05. The Bonferroni-Holm method was used to control the maximum experimentwise error rate for multiple testing [47]. Five variables were considered in this process: HI, CI, DT, the sum of all calculated EAR from the dose volume histograms EAR_sum_^plan^, and the sum of the EAR calculated from the PD measurements EAR_sum_^PD^. Differences in the application of VMAT and IMRT were investigated secondary with a significance level of 0.05 without corrections for multiple testing.

## Results

### Plan quality

**Fig. 3:**
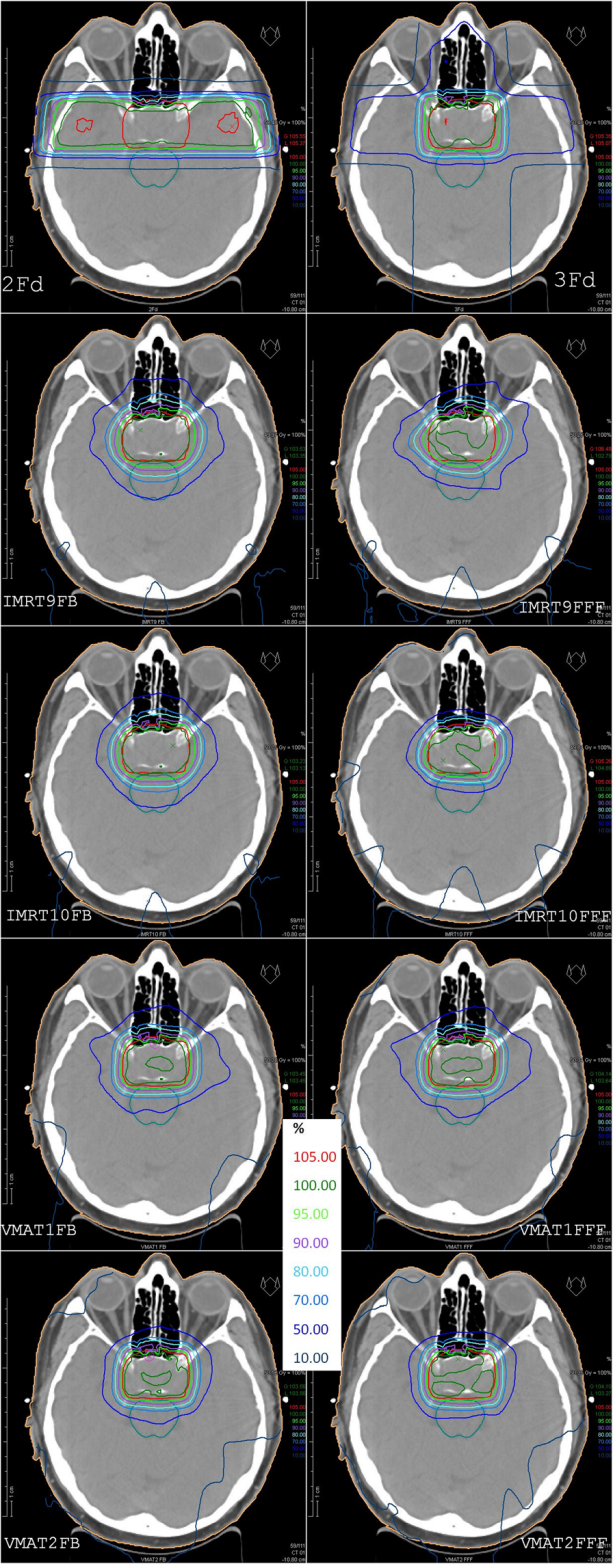
Isodose distributions in the isocenter plane for one representative patient. The first row shows the classical 2 and 3 field techniques, below in the left column plans with FB mode, in the right column the FFF plans. From 2nd row to bottom: coplanar IMRT, non-coplanar IMRT, coplanar VMAT, non-coplanar VMAT. PTV in red and brain stem in cyan are made visible

Nearly all plans ended the optimization process with an average dose in the PTV in the required interval as described in the section “treatment planning” in material and methods. Only one plan (VMAT FB) failed 0.2 Gy below the required minimum value. All other treatment plan objectives have been met in all plans except of the chiasm. This objective has slightly been violated by nearly all plans as the chiasm has been part of the PTV. The maximum value of D_2%_^Chiasm^ was 1.7% above the limit in Table 1. Fig. 3 demonstrates the dose distribution of all calculated plans in the isocenter plane for one representative patient.

**Fig. 4:**
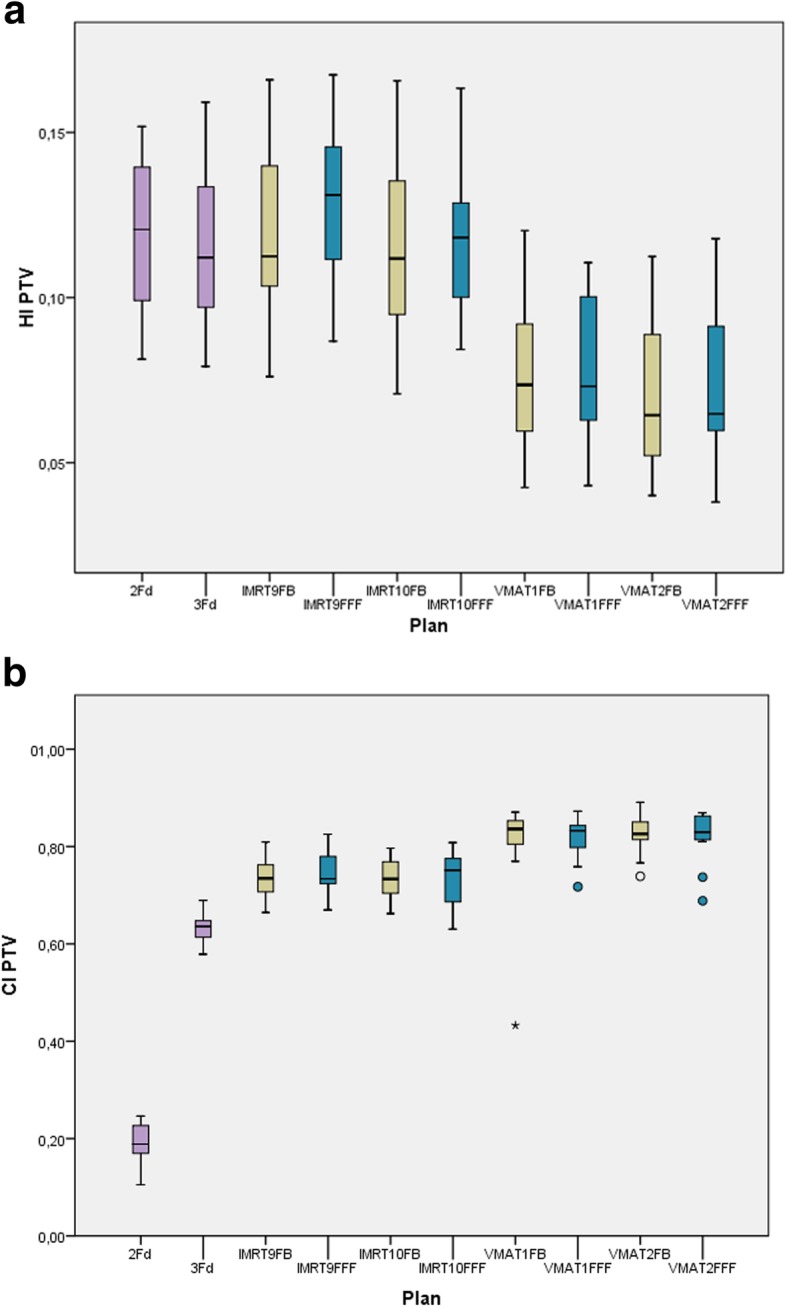
HI (above) and CI (below) as boxplots for all plans: The boxes indicate the inner quartiles, the whiskers the outer quartiles. Outliers and extreme values are indicated by circles and asterisks

Fig. 4 shows the indexes HI and CI. No statistically significant difference has been found for FB and FFF. HI is equivalent for the classical 2Fd and 3Fd techniques and all IMRT plans, but significantly improved for all VMAT plans. CI is lowest for 2Fd plans, shows all IMRT variants on one level and all VMAT variants again improved on a significantly higher level.

**Fig. 5:**
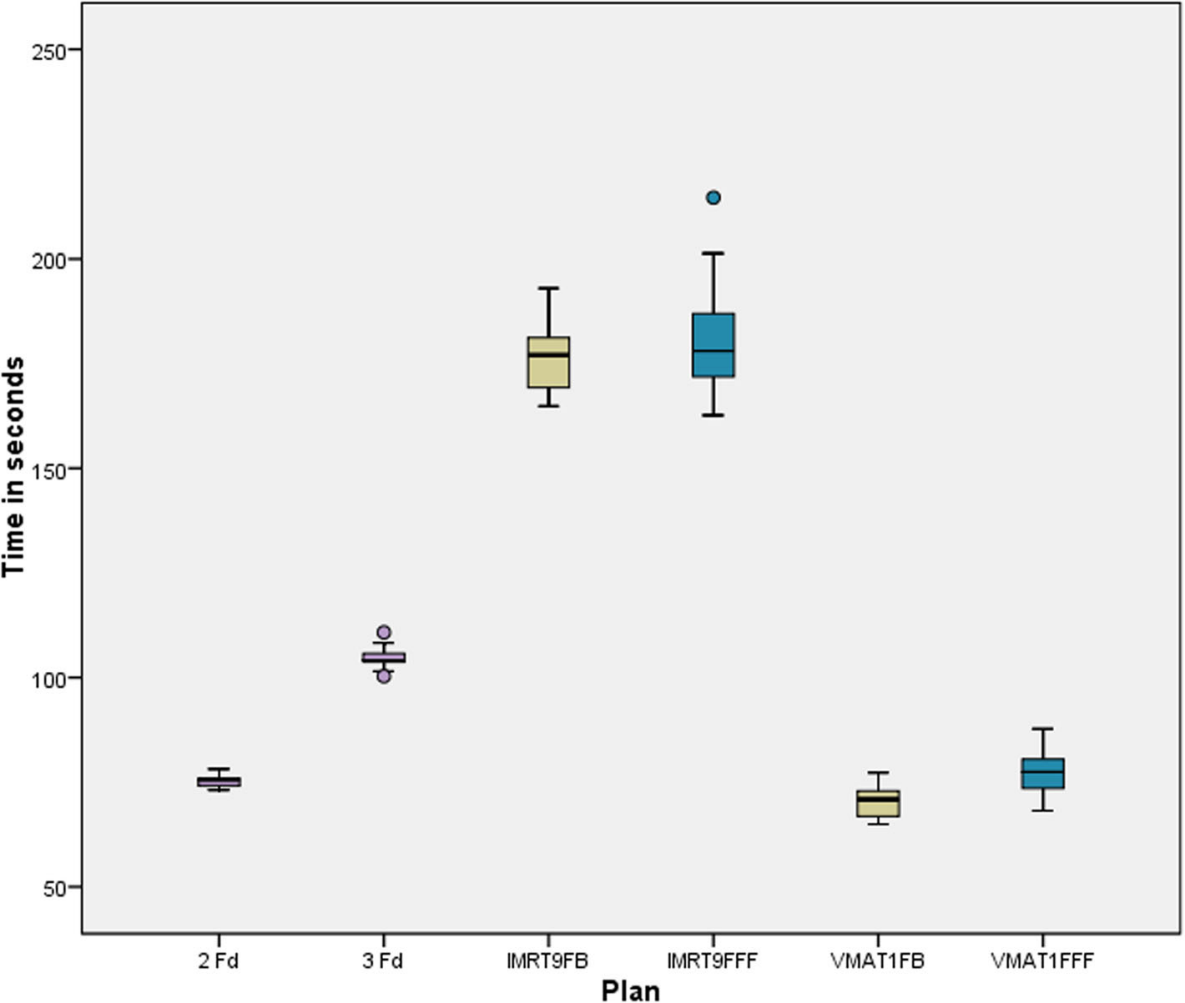
Delivery time for the different plan groups from pressing the start button to the last beam off

In Fig. 5 the delivery time DT is presented for the coplanar plans. For IMRT the difference of FB and FFF is not significant, for VMAT the FFF mode needs statistically significant more time. However, the difference is small (7 s). VMAT takes about the same time as two opposing fields (2Fd) and is faster than 3Fd and takes less than half the time of IMRT. The non-coplanar plans have not been evaluated in detail. Sample measurements have shown that for our local conditions nearly 2 mins additional time are required to enter the treatment room and arrange gantry and table position.

### Secondary malignancy risk

**Fig. 6:**
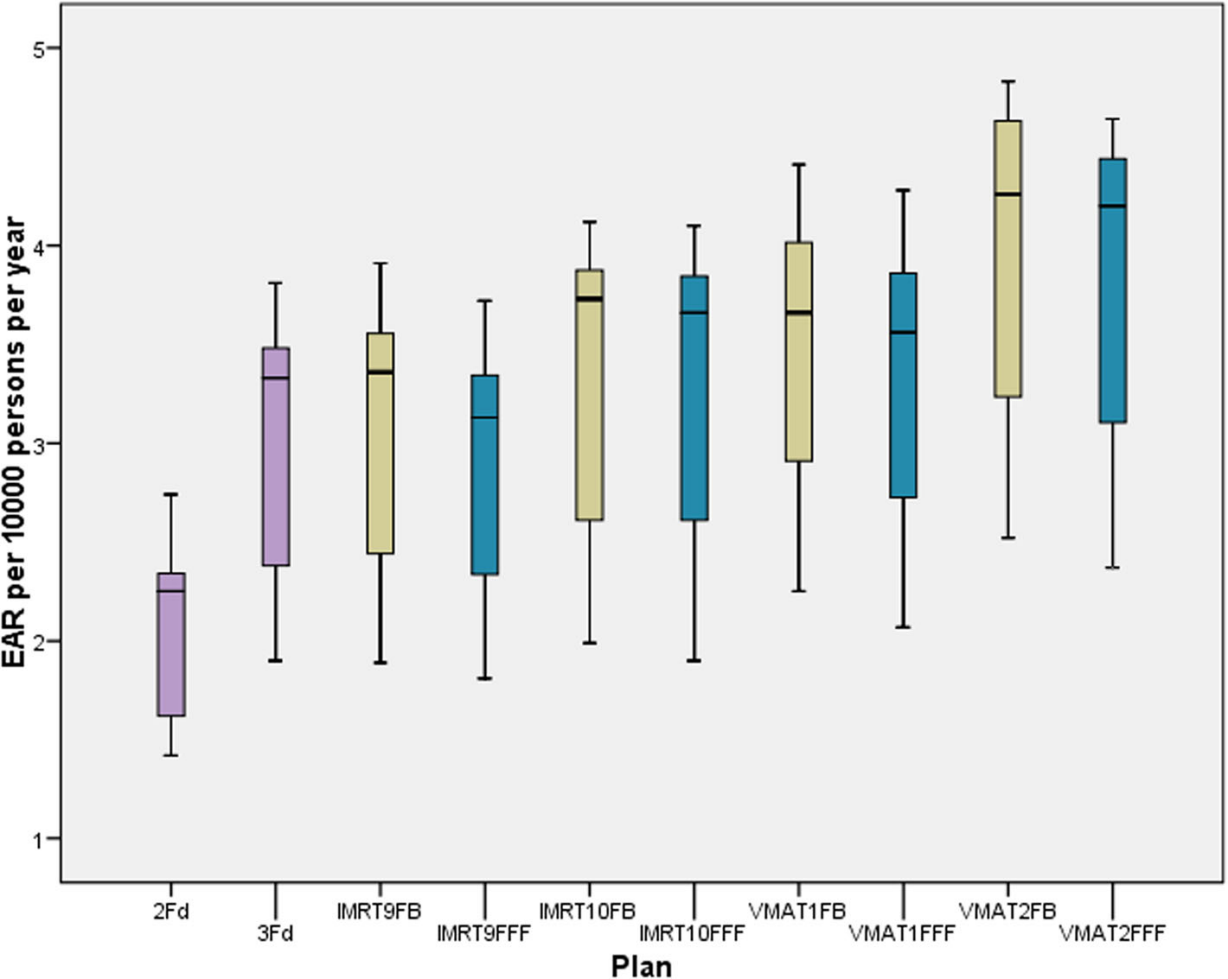
EAR for secondary brain cancer calculated by the mechanistic model, dependent on the different techniques. FFF (blue) is statistically significant lower than FB (yellow)

Fig. 6 illustrates the EAR for secondary brain cancer depending on the technique and mode for the mechanistic model. The other two models are not shown in the figure to gain more clarity. For fluence modulating techniques the values for the bell-shaped model were about 10% higher, for the plateau model about 8% higher than for the mechanistic model. For the 2Fd and the 3Fd technique the differences between the models were up to 19%, but again the bell-shaped model above the plateau model. The reduction of EAR by application of FFF instead of FB is statistically significant. Although the difference between both groups is very small, the significance of the statistics can be explained by the pairing of the samples in the Wilcoxon test: for all 11 pairings, the value for FFF was lower than for FB. The lowest risk is achieved by the simplest technique (2Fd). The differences between the three models are small. The non-coplanar techniques IMRT10 and VMAT2 create a higher risk than the coplanar techniques.

**Fig. 7:**
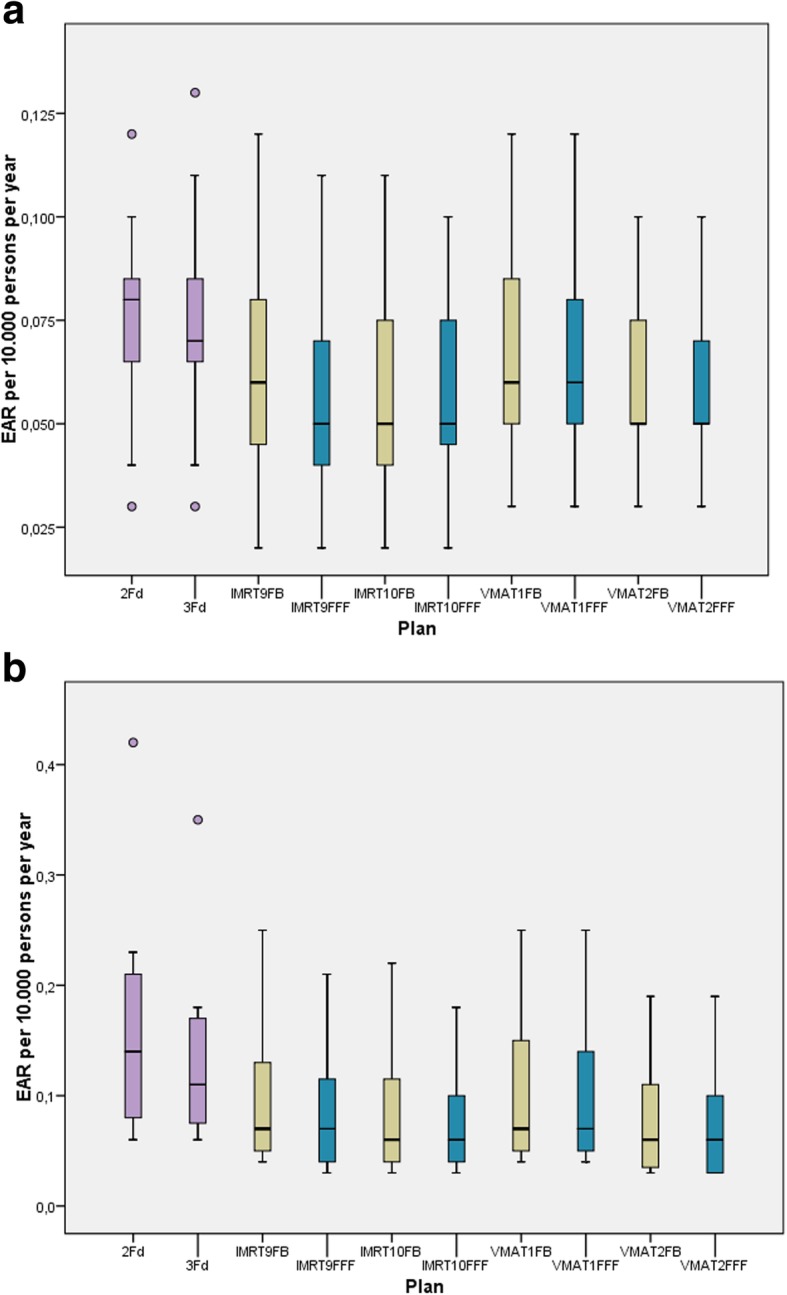
**a**. EAR for secondary bone sarcoma dependent on the different techniques. **b**. EAR for secondary sarcoma of soft tissue dependent on the different techniques

Fig. 7 a and b show the EAR for secondary sarcoma. The risk is one magnitude smaller than for secondary cancer. It is very similar for all techniques. There is nearly no difference between FB and FFF.

**Fig. 8:**
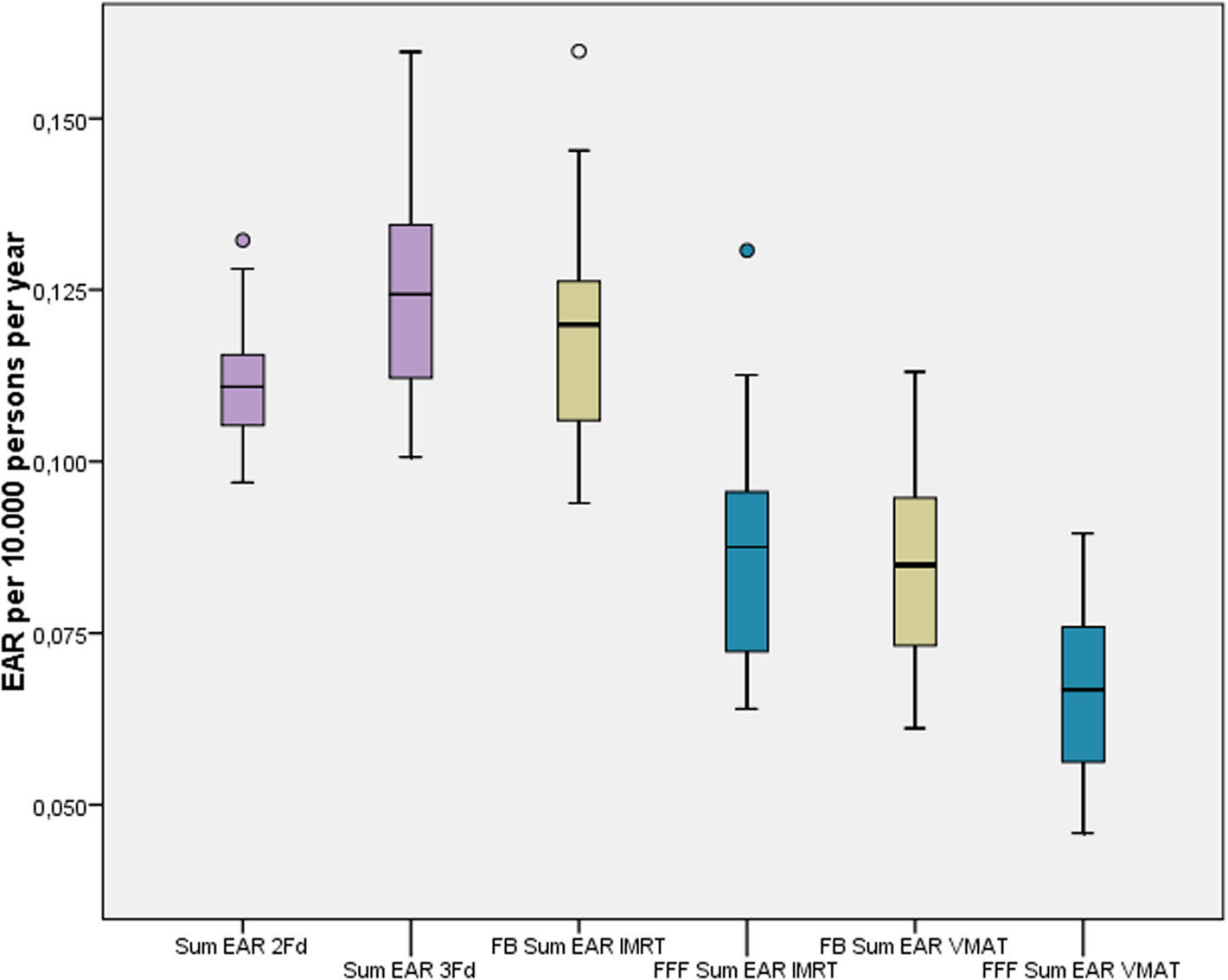
Summed EAR for secondary carcinoma at the peripheral organs thyroid and esophagus. FFF (blue) is statistically significant lower than FB (yellow)

The EAR for secondary cancer in the periphery (thyroid and esophagus) has been derived directly from the PD measurements. In both organs we took the point dose as representative for the whole organ for our calculations as a simple approach. Sample measurements for the non-coplanar techniques resulted in much higher doses with factors from 6 to over 100 and were aborted. The EAR at the esophagus was one magnitude smaller than at the thyroid. For the sake of simplicity we added up both values. The corresponding boxplots are given in Fig. 8. The risk has been reduced statistically significant by the application of FFF mode. VMAT caused the lowest dose and therefore the lowest risk of all techniques.

### Plan verification

The previous results of the plan quality and the secondary malignancy risk demonstrated no benefit for the non-coplanar techniques. Therefore, we verified the coplanar plans only. All plans except of one IMRT plan fulfilled the gamma value acceptance criteria. That means that 95% of the pixels were within the tolerance level mentioned in the material and methods section.

## Discussion

### Plan quality

No rescaling of the dose to the PTV has been performed after the optimization as described in the section material and methods. The failure of reaching the required dose interval for the PTV of one plan could easily be corrected in clinical routine by a rescaling of the MU. The remaining treatment plan objectives would also have been observed after rescaling, except of the chiasm. Choosing other weights for the corresponding objectives of PTV and chiasm might have avoided this little deviation but would probably have impaired the values for HI and CI. Goldsmith et al. [48] assume a tolerance dose of 54 Gy for radiation-induced optic neuropathy when applied in fractions of 1.8 Gy. Therefore, our values were found acceptable. An improved CI is an indicator of a conformal high dose area and therefore of reduced risk for adverse reactions in the organs at risk and unspecified tissue.

Consideration and observance of the dose volume objectives is a main criterion for the clinical acceptability of a treatment plan. The dose limits were taken from published recommendations and resulting risks for the patient were regarded tolerable. No further ranking has been derived from actually achieved mean dose values below the limits. However, in individual cases, e.g. pretreated patients, further dose reduction in a selected organ at risk might be accomplished by another treatment technique, setting, or mode.

The results regarding the plan quality cannot simply be generalized to other equipment or tumors. Dobler et al. found no difference in the application of IMRT or VMAT, FB or FFF for patients with hypopharynx/larynx carcinoma [15]. Alvarez-Moret et al. stated in their study about pediatric patients with ependymoma comparable HI and CI for VMAT FB and FFF, but IMRT FFF superior to IMRT FB [12]. Similarly were the results in an investigation about patients with localized prostate cancer [18].

The 2Fd and 3Fd plans were generated using a 5 mm margin around the PTV. A larger margin probably would improve the HI but also decrease the CI and increase the volume of high dose to unspecified tissue and organs at risk.

The reduction of delivery time in the application of VMAT compared to IMRT was also found in the same order for other entities [12, 18, 49], using the same equipment. Most comparisons of FB and FFF in these studies and also [29] found shorter or statistically not significantly different DTs for FFF. Treutwein et al. [18] discussed that additional MU are required to compensate the profile fall off which also takes additional time. And the potential higher dose rate cannot be exploited between all control points, because the gantry speed and also the speed of the collimating elements are limiting factors [15]. In the present case these influences lead to a slight increase in the DT which is of no clinical importance. This result confirms that findings for other entities cannot simply be transferred to all applications. This has also been stated by Dobler et al. [15] who could not confirm all advantages found for the re-irradiation of spinal column metastasis [49] in their study about the treatment of hypopharynx and larynx carcinoma with the same equipment.

It might be surprising that VMAT can compete with 2Fd technique regarding the DT. However, there is one switching off and on the beam less and there is no inactive rotation time needed.

### Secondary malignancy risk

Schneider et al. [40] presented plots of the EAR as a function of the dose. The plot for the brain and central nervous system shows all three models close together. Although these plots end at a maximum dose of 40 Gy, it seems natural that our results which are based on this study, have also similar results for the three models. Fractionation and recovery show only little influence in the EAR. On the other hand there seems to be a dependency on the irradiated volume. The risk increases from 2Fd over 3Fd, IMRT9, up to the non-coplanar VMAT2 technique.

The excess risk of sarcoma has been found one magnitude smaller than for carcinoma. This is in accordance to the data of Preston et al. [41] of the A-bomb survivors. Schneider et al. [40] concluded from data of radiotherapy patients that the risk might be at comparable magnitude for therapeutic doses. This has not been confirmed for our conditions.

It has been described in the background section that other researchers found reduced PD when applying FFF instead of FB which has been explicated by the missing photon scatter from the flatness filter. Most of these works confined to the documentation of the measured dose. In the present work we calculated the resulting EAR. To our knowledge, similar calculations have only been performed by Murray et al. [17] for a small sample of three patients with early prostate cancer and Alvarez-Moret et al. [12] in a study of pediatric patients with ependymoma. Both confirmed a slightly lower EAR for FFF. The difference between FB and FFF in our study is statistically significant. However, comparing the scales in Figs. 6 and 8 we find the risk at the periphery some magnitude smaller than in the treated region. The magnitude of this ratio is in accordance with the outcomes of Murray et al.

The calculated risks are based on mathematical models and are not directly derived from clinical results. Furthermore, they represent only a part of the risks to which patients with pituitary adenomas are exposed in radiotherapy. Therefore, our results can only support a decision for a specific technique but other factors must be considered.

### Plan verification

The successful plan verifications show that there is no technical problem in the application of FB and FFF, IMRT and VMAT plans. This will probably also be true for the non-coplanar techniques. A hybrid plan verification with original couch angles would not have been possible with our equipment: To avoid the irradiation of the array electronics the patient table could not have been turned to the original angle. As we could not demonstrate any benefit for non-coplanar plans for our standard setup we passed on their verification. However, in individual cases, e.g. pretreated persons, their application can be reasonable.

## Conclusion

It has been shown that in the treatment of pituitary adenomas plans which use the FFF mode are of equal quality as FB plans regarding the homogeneity, the conformity, and the dose to the organs at risk. FFF plans are superior in the respect of secondary malignancy induction. VMAT is the fastest advanced technique, on the same level as opposing fields. Non-coplanar techniques showed no benefit for the investigated parameters but need much more time for couch rotation. Opposing fields cause the lowest secondary brain cancer risk but have the lowest conformity.

For most patients we regard VMAT the better choice than IMRT due to slightly improved HI and CI and clearly shortened treatment times. We regard the risk for secondary malignancies as a minor effect which is of the same magnitude for all techniques and modes. However, it can be used as a subordinated criterion with reduced risk using FFF. Taking all results into account coplanar VMAT FFF seems the most preferable technique for the treatment of pituitary adenomas with the given equipment.

## Abbreviations

2Fd: Two opposing fields
3Fd: Three field technique
CI: Conformity index
CTV: Clinical target volume
DT: Delivery time
EAR: Excess absolute risk
FB: Flattened beam (with flatness filter)
FFF: Flattness filter free
GTV: Gross target volume
HI: Homogeneity index
IMRT: Intensity modulated radiotherapy
MU: Monitor units
PD: Peripheral dose
PTV: Planning target volume
RED: Risk equivalent dose
VMAT: Volumetric modulated arc therapy
